# Association of radiation dose intensity with overall survival in patients with distant metastases

**DOI:** 10.1002/cam4.4304

**Published:** 2021-09-30

**Authors:** Johnny Kao, Mark K. Farrugia, Samantha Frontario, Amanda Zucker, Emily Copel, John Loscalzo, Ashish Sangal, Boramir Darakchiev, Anurag Singh, Symeon Missios

**Affiliations:** ^1^ Department of Radiation Oncology Good Samaritan Hospital Medical Center West Islip New York USA; ^2^ The Cancer Institute at Good Samaritan West Islip New York USA; ^3^ Department of Radiation Medicine, Roswell Park Cancer Institute Buffalo New York USA; ^4^ Symptom Management and Supportive Care Service, Good Samaritan Hospital Medical Center West Islip New York USA; ^5^ Long Island Brain and Spine West Islip New York USA

**Keywords:** metastatic, patient selection, radiotherapy, survival

## Abstract

**Background:**

Patients with metastatic cancer referred to radiation oncology have diverse prognoses and there is significant interest in personalizing treatment. We hypothesized that patients selected for higher biologically equivalent doses have improved overall survival.

**Methods:**

The study population consists of 355 consecutive adult patients with distant metastases treated by a single radiation oncologist from 2014 to 2018. The validated NEAT model was used to prospectively stratify patients into four distinct cohorts. Radiation dose intensity was standardized using the equivalent dose in 2 Gy fractions (EQD2) model with an *α*/*β* of 10. Radiation dose intensity on survival was assessed via Cox regression models and propensity score match pairing with Kaplan–Meier analysis.

**Results:**

The median survival was 9.3 months and the median follow‐up for surviving patients was 18.3 months. The NEAT model cohorts indicated median survivals of 29.5, 11.8, 4.9, and 1.8 months. Patients receiving an EQD2 of ≥40 Gy had a median survival of 16.0 months versus 3.8 months for patients receiving an EQD2 of <40 Gy (*p* < 0.001). On multivariable analysis, performance status, primary tumor site, radiation dose intensity, albumin, liver metastases, and number of active tumors were all independent predictors of survival (*p* < 0.05 for all). Propensity score matching was performed for performance status, albumin, number of active tumors, primary tumor site, and liver metastasis, finding higher EQD2 to remain significantly associated with improved survival within the matched cohort (*p* = 0.004).

**Conclusion:**

Higher radiation dose intensity was used in patients with better prognosis and was associated with improved survival for patients with metastatic disease.

## Graphical abstract

Personalized radiotherapy treatment options for patients with metastatic is of significant interest and prompted investigation into higher biological equivalent doses to see if there was increased survival. Higher dose intensity was used for patients with better progresses and was associated with improved survival with metastatic diseases.

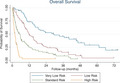

## 1 INTRODUCTION

Patients with metastatic cancers referred to radiation oncology are heterogeneous in terms of both presentation and prognosis.^1^ Accurately predicting survival is difficult as evidenced by the recently published SCORAD trial demonstrating that 37% 8‐week mortality among patients with a life expectancy of >8 weeks.^2^ Recent efforts have markedly improved prognostication of patients with metastatic cancer in the radiation oncology setting.^3^, ^4^, ^5^ Improved prognostication holds promise to deliver more optimized, personalized radiation treatment regimens for patients with metastatic cancer. For patients nearing the end of life, accumulating evidence suggests that radiation therapy should be abbreviated or even withheld while focusing on supportive care and symptom management.^6^, ^7^ On the other extreme, recent randomized trials suggest an overall survival benefit for intensified radiation therapy for favorable prognosis patients with oligometastases.^8^, ^9^

Over the past several years, our group has developed and validated a prognostic tool to predict survival for patients with metastatic cancer that was agnostic to treatment received.^1^, ^5^ Accurately predicting survival for metastases across disease sites is particularly relevant to community practice in contrast to academic centers where subspecialization is more common.^10^ After analyzing 29 candidate prognostic factors, 4 prognostic factors independently predicted survival on multivariable analysis.^1^ The variables contributing to the NEAT model, include number of active tumors on whole body imaging (1 to 5 vs. ≥6), Eastern Cooperative Oncology Group (ECOG) performance status (0–1 vs. 2 vs. 3–4), albumin (≥3.4 vs. 2.4 to 3.3 vs. <2.4), and primary tumor (breast, prostate, kidney vs. other).^1^ Further analyses suggested that liver tumors, recent hospitalization and neutrophil to lymphocyte ratio may provide further prognostic information.^5^, ^11^ In clinical practice, implementation of the NEAT model increasingly informed radiation treatment decisions over time such that higher radiation dose intensity was more frequently used in better prognosis patients. Given that accurately predicting survival in this population is challenging, we hypothesized that patients selected for more intensive radiation schedules is a previously unmeasured surrogate for better prognosis.

## 2 MATERIALS AND METHODS

The institutional review board approved this retrospective cohort study. The study population consists of 392 consecutive adult patients with metastatic cancer referred to a single radiation oncologist at a community hospital with a comprehensive community cancer program from 1 January 2014 to 31 December 2018. This analysis included 73 patients with metastatic cancer enrolled on a prospective clinical trial evaluating the accuracy of clinician predictions from 14 October 2016 to 8 December 2017. For patients who received multiple courses of radiotherapy, this analysis was limited to the first radiation oncology consultation that occurred between 2014 and 2018.

The starting date was selected to reflect the availability of technology that enabled modern image‐guided radiation therapy, stereotactic body radiation therapy, and frameless stereotactic radiotherapy. Patient records were reviewed to extract patient, disease and treatment characteristics using both EPIC and Aria electronic medical record systems. Relevant information collected prior to radiation oncology consultation included age, gender, ECOG performance status, baseline serum albumin, baseline neutrophil to lymphocyte ratio, primary tumor site, number of active tumors on whole body imaging (computed tomography [CT] of the chest, abdomen, and pelvis ± brain imaging and/or positron emission tomography/CT), involved metastatic sites, number of systemic regimens for metastatic cancer and hospitalization within 3 months of radiation oncology consultation. Based on this information, patients were prospectively classified into the four‐tiered NEAT model as previously described.^1^

### 2.1 Treatment

Treatment consisting of systemic therapy, radiation therapy, and surgery was administered at the discretion of the team of treating oncologists. Radiation equipment included a Varian Truebeam equipped with cone beam CT and a 6‐degree of freedom robotic couch, a Varian 21EX linear accelerator, a Nucleotron remote afterloader for high dose rate brachytherapy and four‐dimensional CT simulation capability was available. Radiation dose, number of fractions, number of isocenters, and location of metastasis were recorded for all treated sites. Assuming an *α*/*β* of 10, the equivalent dose in 2 Gy fractions (EQD2) methodology was performed to calculate a biologically equivalent dose. When multiple sites were treated, the highest EQD2 dose was analyzed. An EQD2 of ≥40 Gy was considered high dose, since this level included the vast majority of patients treated with stereotactic radiotherapy (Table S1).

### 2.2 Statistical analysis

Statistical evaluation was performed using Stata 13.1 and R. Survival is measured from the date of the consultation to the date of death or last follow‐up. Survival was ascertained through clinical follow‐up, electronic medical record search, and follow‐up phone calls to patients and family members. Overall survival was calculated using the Kaplan–Meier method. Differences in patient characteristics for patients with low and high EQD2 radiation were assessed using the Pearson’s chi‐squared test. Differences in survival by radiation dose were assessed using the log‐rank test with a *p*‐value of 0.05 considered statistically significant. To adjust for potential confounding, covariates with a *p*‐value of <0.05 in univariate analysis were included in a multivariate Cox regression model.

Propensity score matching was performed for variables found to be statistically significant on Cox multivariable analysis. Propensity score matching for ECOG (0–1 vs. 2+), primary tumor (favorable vs. unfavorable), albumin (≥3.4 vs.<3.4), number of active tumors (1–5 vs. >5), and liver metastasis was performed on the 291 patients with known albumin and neutrophil to lymphocyte ratio using a 1:1 ratio and nearest neighbor method, caliper length of 0.1 in R (version 4.0.2) with package MatchIt version 3.0.2. Based on these selection criteria, 76 matched pairs were generated with well‐balanced covariates (*p* = 1.0) (Table S2).

## 3 RESULTS

### 3.1 Patient characteristics and radiation therapy

Among 392 consecutive patients with metastatic cancer referred to a single radiation oncologist, 355 patients (91%) were treated with radiation therapy. This analysis focuses on the patients receiving radiation with a median survival was 9.3 months (range 0.2 months to 74.3+ months). The median follow‐up for surviving patients was 18.3 months. The median age was 68 years (range 23–96) and 50% were male and 50% were female. Patient characteristics are summarized in Table 1. The most common sites of distant metastases were bone (54%), lung and pleura (33%), distant lymph nodes (30%), brain (26%), liver, (22%), and adrenal (7%). The most common sites treated were bone metastases (41%), primary tumor ± regional nodes (36%), brain metastases (26%), lung metastases (7%), and distant lymph nodes (7%) (Table S3).

**TABLE 1:** Median and 12‐month survival stratified by patient characteristics

	Number (%)	*p*	Median survival (months)	12‐month survival
Overall population			9.3	41.9%
Age		0.23		
<60	80 (22.5%)		9.3	40.3%
≥60	275 (77.7%)		9.2	42.5%
Gender		0.43		
Male	179 (50.4%)		8.6	41.5%
Female	176 (49.6%)		9.3	42.3%
ECOG performance status		<0.001		
0–1	178 (50.1%)		15.4	56.3%
2	108 (30.4%)		6.4	35.6%
3–4	69 (19.5%)		2.5	8.4%
Albumin		<0.001		
≥3.4	153 (43.1%)		14.4	54.7%
2.4–3.3	128 (36.1%)		5.5	24.5%
<2.4	24 (6.8%)		2.5	4.9%
Unknown	50 (14.1%)		17.0	60.8%
Neutrophil to lymphocyte ratio		0.001		
≤4.0	131 (36.9%)		12.4	50.3%
>4.0	177 (49.9%)		5.6	30.4%
Unknown	47 (13.2%)		19.0	60.6%
Primary tumor site				
Lung	152 (42.8%)		7.0	35.7%
Prostate	39 (11.0%)		28.0	68.6%
Breast	38 (10.7%)		9.3	38.4%
Colorectal	19 (5.4%)		8.8	39.0%
Uterus	17 (4.8%)		16.3	55.8%
Esophagus/gastric	14 (3.9%)		4.4	15.5%
Unknown primary	13 (3.7%)		6.8	29.4%
Melanoma	12 (3.4%)		1.8	25.0%
Kidney	8 (2.3%)		18.4	62.5%
Pancreatic/hepatobiliary	8 (2.3%)		15.5	57.1%
Other (ovary, cervix, sarcoma, skin, bladder, pleura, head and neck, vulva)	35 (9.9%)		12.4	52.2%
Favorable primary site		0.003		
Breast, prostate, or kidney	85 (23.9%)		18.4	54.3%
Others	270 (76.1%)		7.0	37.9%
Number of active tumors		<0.001		
1–5	106 (29.9%)		22.4	66.1%
≥6	249 (70.1%)		6.1	31.0%
Liver metastases		<0.001		
Yes	80 (22.5%)		4.3	13.7%
No	275 (77.5%)		11.8	49.2%
Bone only metastases		<0.001		
Yes	80 (22.5%)		22.4	67.2%
No	275 (77.5%)		6.8	34.5%
Hospitalization within the prior 3 months		<0.001		
Yes	204 (57.5%)		5.5	28.8%
No	151 (42.5%)		15.7	58.3%
Prior systemic therapy regimens for distant metastases		0.44		
0–1	297 (83.7%)		9.3	42.1%
≥2	58 (16.3%)		8.8	40.9%
Radiation dose intensity (EQD2)		<0.001		
≥40 Gy	195 (54.9%)		16.0	57.4%
<40 Gy	160 (45.1%)		3.8	22.2%

Abbreviations: ECOG, Eastern Cooperative Oncology Group; EQD2, equivalent dose in 2 Gy fractions.

### 3.2 Predictors of survival on univariate analysis

**FIGURE 1:**
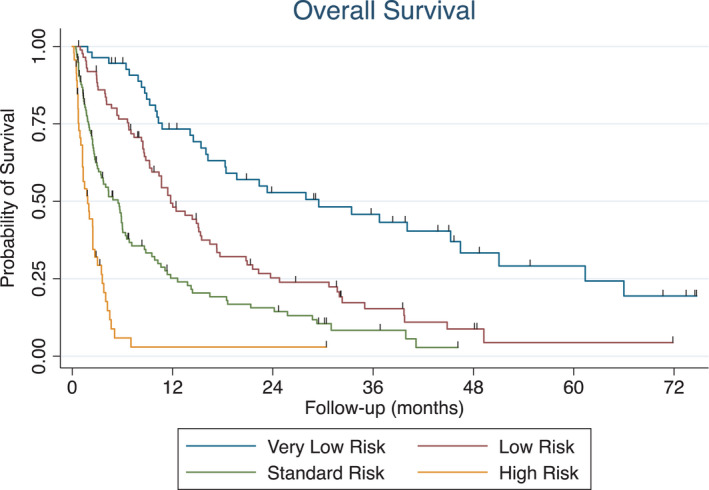
Overall survival stratified by NEAT risk group

Various patient characteristics and their impact on survival are summarized in Table 1. Poor performance status, low serum albumin, high neutrophil to lymphocyte ratio, widespread disease extent (>5 active tumors), liver metastases, and recent hospitalization all predicted for worse survival (*p* < 0.05) for all. Age and gender were not predictive of survival (*p* > 0.05) for all. For primary tumor site, breast, prostate, or kidney tumors were considered favorable sites (*p* < 0.05). The NEAT model separated patients into four distinct groups with median survivals of 29.5, 11.8, 4.9, and 1.8 months, respectively (Table 2; Figure 1). Analysis of number of isocenters and metastasis site treated and survival is shown in Table S3.

**TABLE 2:** Median and 12‐month survival stratified by the NEAT risk score

NEAT group	Number (%)	Median survival (months)	12 month survival (95% confidence interval)
Very low risk	55 (18%)	29.5	73.3% (59.1–83.3)
Low risk	87 (29%)	11.8	49.4% (38.2–59.6)
Intermediate risk	117 (38%)	4.9	24.9% (16.8–34.0)
High risk	46 (15%)	1.8	2.9% (0.2–12.9)

### 3.3 Association of radiation dose intensity on survival

Among patients with NEAT very favorable, favorable, standard, and unfavorable risk disease, the percentage of patients receiving an EQD2 of ≥40 Gy was 76%, 68%, 43%, and 20%, respectively.

**FIGURE 2:**
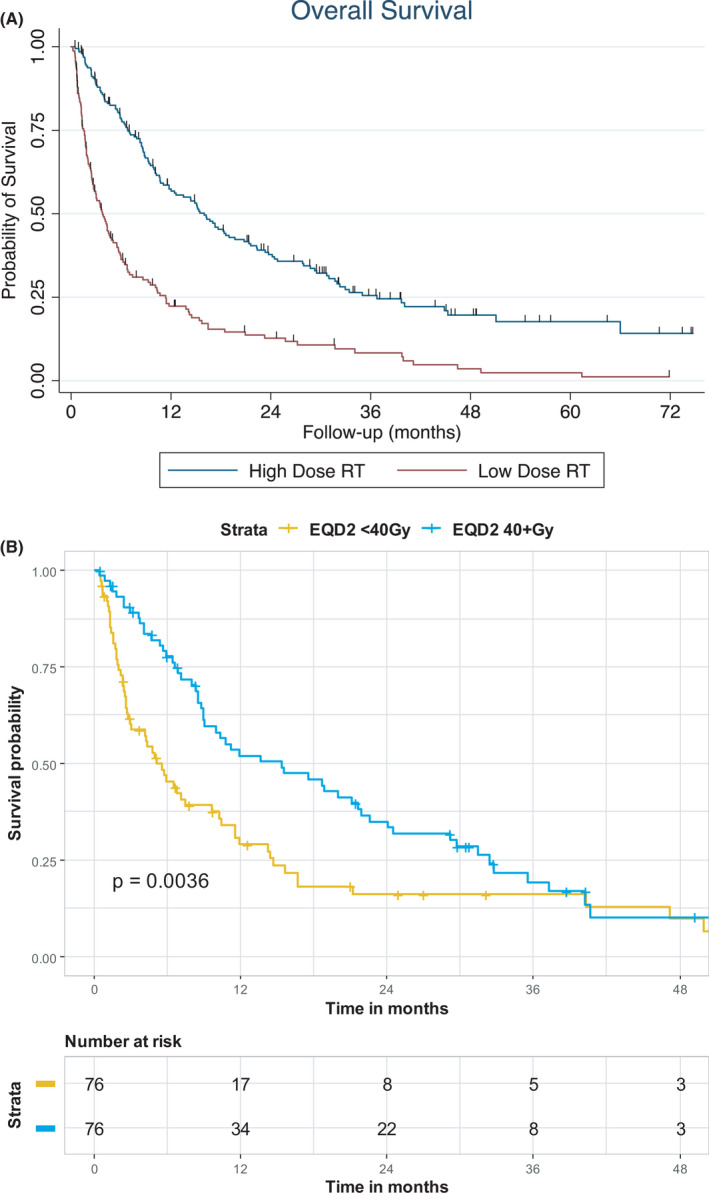
(A) Effect of radiation dose intensity (equivalent dose in 2 Gy fractions [EQD2] >40 Gy vs. ≤40 Gy) on overall survival for the entire cohort. (B) Effect of radiation dose intensity (EQD2 >40 Gy vs. ≤40 Gy) on overall survival among 152 patients using propensity score matching

Patients receiving an EQD2 of ≥40 Gy had a median survival of 16.0 months compared to 3.8 months for patients receiving an EQD2 of <40 Gy, *p* < 0.001 (Table 1; Figure 2). The 15 most common dose fractionation schedules accounting for 70% of patients are described in Table S1. The low‐dose cohort included 37 patients who received <80% of the prescribed dose with a median survival of 1.3 months. Patient and treatment characteristics for patients treated with high‐ or low‐dose intensity radiation are shown in Table S4.

### 3.4 Multivariable analysis and propensity score matching

On multivariable analysis, performance status, primary tumor site, radiation dose intensity, albumin, liver metastases, and number of active tumors were all independent predictors of survival (Table 3). Recent hospitalization, bone only metastases, and neutrophil to lymphocyte ratio were not predictive. Radiation dose intensity remained statistically significant even when patients with incomplete courses of radiation were excluded from multivariable analysis. Including treatment variables such as number of isocenters, brain metastases, and lung metastases did not alter the findings of the multivariable analysis (Table S5).

**TABLE 3:** Cox multivariable analysis of predictors of overall survival

Variable	Hazard ratio	95% Confidence interval	*p* value
ECOG performance status (0–1 vs. 2 vs. 3–4)	1.67	1.37–2.03	<0.001
Radiation dose intensity (high vs. low)	1.97	1.44–2.70	<0.001
Serum albumin (≥3.4 vs. 2.4 to 3.3 vs. <2.4)	1.48	1.16–1.87	0.001
Tumor site (breast, kidney or prostate vs. other)	1.76	1.21–2.55	0.003
Liver metastases (no vs. yes)	1.54	1.10–2.14	0.011
Number of active tumors (1–5 vs. ≥6)	1.53	1.07–2.18	0.020
Bone only metastases (yes vs. no)	1.38	0.91–2.09	0.129
Hospitalized within prior 3 months (no vs. yes)	1.25	0.91–1.71	0.169
Neutrophil to lymphocyte ratio (≤4.0 vs. >4)	1.16	0.87–1.55	0.305

Abbreviation: ECOG, Eastern Cooperative Oncology Group.

On propensity score matching analysis, the median survival was 15.4 months for EQD2 of ≥40 Gy versus 5.1 months for EQD2 of <40 Gy (*p* = 0.0036).

### 3.5 Evidence of improved prognostication impacting patient selection and outcome over time

An exploratory analysis was performed to determine whether patient selection for higher EQD2 doses improved over time. The rationale for using 2017 as a cutpoint was twofold. In late 2016, our group validated the published NEAT model, launched a clinical trial based on NEAT predictions and began routinely using NEAT predictions in clinical practice. Second, immunotherapy was more widely used for lung cancer in the later cohort.

**FIGURE 3:**
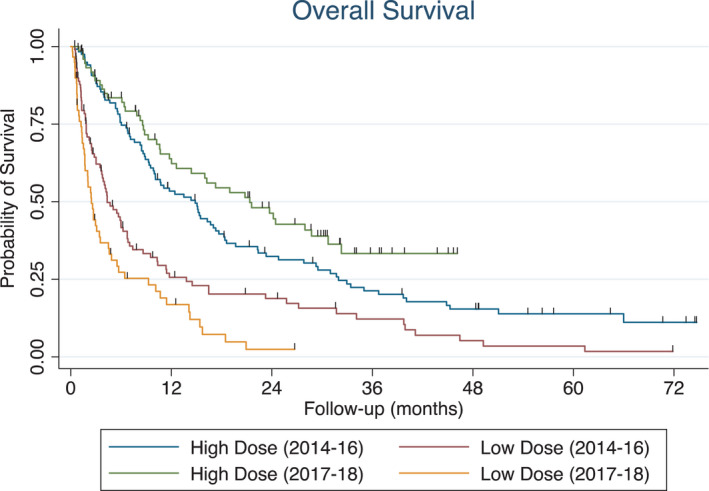
Differential effects of radiation dose intensity on survival from 2014 to 2016 versus 2017 to 2018

Overall, patients referred in 2014–2016 had a median survival of 9.3 months versus 9.3 months for patients referred from 2017 to 2018 (*p* = 0.87). For patients referred to radiation oncology from 2014 to 2016, patients treated with high‐dose radiation had a median survival of 14.9 months versus 4.3 months for patients receiving low‐dose radiation. In contrast, patients referred from 2017 to 2018 had a median survival of 21.4 months with high‐dose radiation versus 2.5 months with low‐dose radiation (Figure 3).

## 4 DISCUSSION

This study demonstrates that radiation dose intensity is associated with improved overall survival among patients with metastatic cancers selected for treatment intensification. These patients were felt to have better prognosis and thus preferentially selected for higher‐dose intensity (Table S4). Given the inherent covariance of these variables, the extent that this survival advantage is due to patient selection or treatment effect is unclear, even with multivariable analysis and propensity score matching.

For patients with widespread bone metastases receiving palliative radiotherapy, radiation dose escalation does not improve survival, at least within the dynamic range of 8 Gy in 1 fractions to 30 Gy in 10 fractions corresponding to an EQD2 of 12.0–32.5 Gy.^2^, ^6^

There are accumulating data from multiple randomized controlled trials demonstrating improved survival with intensified radiation for patients with extracranial oligometastases, limited brain metastases, and prostate cancer with limited bone metastases.^8^, ^9^, ^12^ Whether there is a benefit of radiation dose escalation for other subgroups that comprise the majority of patients with metastatic cancer referred to radiation oncology is worthy of investigation. To follow‐up on this hypothesis generating analysis, our group is currently enrolling patients on a randomized controlled trial comparing dose‐intensified stereotactic body radiotherapy to 16–26 Gy in 1 fraction versus standard palliative radiotherapy in 1–10 fractions for patients with extracranial metastases (NCT04068649). Dose intensified but volume reduced radiation could have benefits beyond survival, such as reducing neurocognitive failure and alopecia by substituting stereotactic radiation for whole brain radiotherapy for brain metastases and decreased pain when substituting stereotactic body radiation therapy for palliative radiotherapy for painful bone metastases.^13^, ^14^ In a recent study of patients with metastatic cancer treated at Stanford Cancer Institute from March 2015 to November 2016, 50% of patients were treated with stereotactic ablative radiation.^15^ A large prospective registry study demonstrated 79% overall survival at 2 years for patients selected for stereotactic body radiotherapy based on good performance status, limited metastatic disease and life expectancy of at least 6 months.^16^ Taken together, the concept of dose escalation to EQD2 doses ≥40 Gy for selected patients with metastatic disease is well established but specific selection criteria remain incompletely defined.

Prior work from our group demonstrated the accuracy of existing prognostic models for metastatic cancer in radiation oncology patients, including the Chow, TEACHH, and NEAT models.^5^ The current study provides evidence that radiation therapy treatment decisions are influenced by clinical intuition and judgment beyond the NEAT model alone. For patients referred to radiation oncology from 2017 to 2018, the median survival for patients treated with high‐dose radiation was 21.4 months versus 2.5 months for patients who completed low‐dose radiation (*p* < 0.001). Patients selected for 8 Gy in one fraction or 20 Gy in five fractions had a median survival of <2 months, considerably lower than the median survival of 9.3 months for the entire cohort (Table S1). While work on decision support tools continues rapidly, these data argue for a continued role for a human‐in‐the‐loop to optimize treatment decisions as opposed to relying solely on prognostic models alone.^17^

In the academic setting where clinicians often focus on a small number of disease sites, radiation treatment of metastatic disease is increasingly viewed as a distinct subspecialty.^10^ In contrast, in the community setting, the radiation oncologist maintains continuity of care and needs to expertly administer treatment for both primary and metastatic tumors. For a general practice, prognosis informed management offers promise to streamline and simplify nuanced treatment decisions for patients with metastatic solid tumor. This large series of consecutive patients referred to a single community‐based radiation oncologist provides cross‐sectional context into patient selection that is facilitated by the NEAT model. A proposed radiation decision support algorithm is presented in Table 4.

**TABLE 4:** A proposed prognosis‐informed algorithm for personalized radiation treatment prescriptions

NEAT group	Median survival (months)	Radiation plan
Very low risk	30	High dose (stereotactic radiation or curative intent conventional radiation [RT] up to 33 fractions)
Low risk	12	High dose (stereotactic radiation) or standard dose RT (10–15 fractions)
Intermediate risk	5	Extracranial: low dose RT (5–10 fractions) Cranial: stereotactic radiation (1–5 fractions) if technically feasible
High risk	2	No RT or very short course RT (1–5 fractions)

Abbreviation: RT, radiation therapy.

In this series of patients referred from January 2014 to December 2018, the median survival for patients receiving radiation therapy for metastatic disease was 9.3 months compared to 5.5 months for patients referred from May 2012 to August 2013.^1^ In the more recent era, dose‐intensified radiation for patients with oligometastases and frameless stereotactic radiation in lieu of whole brain radiotherapy for patients with 4–10 brain metastases were increasingly utilized.^18^, ^19^ Significant advances in systemic therapy markedly contributed to outcome, particularly patients with advanced lung cancer.^20^ Therefore, models to predict survival require cycles of continuous improvement. Incorporation of frailty measures, next‐generation sequencing, analysis of very large datasets, and the application of artificial intelligence hold promise to further refine predictive models.^21^

The primary weakness of this study is the retrospective, non‐randomized single institution design with inherent selection biases (43% lung cancer). Including the experience of a single physician limits the generalizability of the results. Moreover, the study population included a large variety of primary tumors and histologies with variable sensitivities to treatment and widely diverging prognoses. In the absence of randomization, it is not possible to definitively conclude that higher biologically equivalent doses translated to improved survival based on treatment effect or whether patients selected for higher doses reflected favorable prognostic characteristics that were unmeasured by the NEAT model. Conversely, patients with historically good prognosis breast and prostate cancers (22% of the total study population) accounted for 43% of patients treated with 30 Gy in 10 fractions due to the perception that higher doses were not necessary to achieve durable palliation.

In contrast to other studies that focused on specific organ involvement, primary tumor types, palliative intent, or oligometastases, this cross‐sectional study attempts to comprehensively analyze a relatively unselected cohort of patients with metastatic disease referred to radiation oncology.

In conclusion, high‐radiation dose intensity defined as EQD2 ≥40 Gy is a surrogate marker for prognosis associated with improved survival for patients with metastatic disease.

## CONFLICT OF INTEREST

J. Kao serves on an advisory board with Astra Zeneca. J. Kao and S. Missios led an educational webinar for Varian. All other authors report no conflict of interests, compensation, ownerships, investments, and leadership positions.

## ETHICAL APPROVAL STATEMENT

Data are collected from Good Samaritan Hospital Medical Center and are devoid of any personal identifiable information.

## Supporting information

Table S1‐S5

## Data Availability

The data that supports the findings of this study are available in the supplementary material of this article.
