# Supplementary material for: Association of radiation dose intensity with overall survival in patients with distant metastases
Source: Cancer Med. 2021 Sep 30;10(22):7934–42. doi: 10.1002/cam4.4304 (PMC8607262; doi:10.1002/cam4.4304)
Supplement: Supplementary file 1 — Table S1‐S5 [file CAM4-10-7934-s001.docx]

| **Radiation Regimen** | EQD2 | Number | Median survival (months) | 12 month survival |
| --- | --- | --- | --- | --- |
| 8 Gy in 1 fraction | 12.0 | 6 | 1.0 | 0.0% |
| 20 Gy in 5 fractions | 23.3 | 12 | 1.7 | 11.1% |
| 30 Gy in 10 fractions | 32.5 | 63 | 9.5 | 37.6% |
| 35 Gy in 14 fractions | 36.5 | 9 | 4.3 | 11.1% |
| 37.5 Gy in 15 fractions | 39.1 | 17 | 5.5 | 22.1% |
| 30 Gy in 5 fractions | 40.0 | 20 | 15.2 | 55.9% |
| 40 Gy in 16 fractions | 41.7 | 15 | 10.1 | 36.4% |
| 27 Gy in 3 fractions | 42.8 | 38 | 10.7 | 46.2% |
| 40 Gy in 10 fractions | 46.7 | 15 | 18.4 | 68.1% |
| 45 Gy in 15 fractions | 48.8 | 8 | 28.8 | 62.5% |
| 50 Gy in 25 fractions | 50.0 | 9 | 11.8 | 44.4% |
| 50 Gy in 20 fractions | 52.1 | 7 | 16.7 | 57.1% |
| 60 Gy in 30 fractions | 60.0 | 9 | 10.7 | 44.4% |
| 50 Gy in 10 fractions | 62.5 | 16 | 24.4 | 74.0% |
| 66 Gy in 33 fractions | 66.0 | 6 | 29.5 | 83.3% |

Supplemental Table 1. Overall Survival by Radiation Dose Regimen

| **Variable** | **EQD2 ≥40 Gy**  (n=76) | **EQD2 <40 Gy**  (n=76) | **p** |
| --- | --- | --- | --- |
| **Age (years)** | Median 68.5 | Median 66.0 | 0.44 |
| **Gender** |  |  | 0.52 |
| Male | 39 (51.3%) | 35 (46.1%) |  |
| Female | 37 (48.7%) | 41 (53.9%) |  |
| **ECOG performance status** |  |  | 1.00 |
| 0-1 | 40 (52.6%) | 40 (52.6%) |  |
| 2 to 4 | 36 (47.4%) | 36 (47.4%) |  |
| **Albumin** |  |  | 1.00 |
| ≥3.4 | 34 (44.7%) | 34 (44.7%) |  |
| <3.4 | 42 (55.3%) | 42 (55.3%) |  |
| **Neutrophil to Lymphocyte Ratio** |  |  | 0.19 |
| ≤4.0 | 30 (39.5%) | 29 (38.2%) |  |
| >4.0 | 46 (60.5%) | 47 (61.8%) |  |
| **Primary tumor site** |  |  |  |
| Breast, Prostate or Kidney | 21 (27.6%) | 21 (27.6%) | 1.00 |
| Other | 55 (72.4%) | 55 (72.4%) |  |
| **Number of Active Tumors** |  |  | 1.00 |
| 1 to 5 | 13 (17.1%) | 13 (17.1%) |  |
| ≥6 | 63 (82.9%) | 63 (82.9%) |  |
| **Liver Metastases** |  |  | 1.00 |
| Yes | 21 (27.6%) | 21 (27.6%) |  |
| No | 55 (72.4%) | 55 (72.4%) |  |
| **Hospitalization within the Prior 3 Months** |  |  | 0.737 |
| Yes | 49 (64.5%) | 47 (61.8%) |  |
| No | 27 (35.5%) | 29 (38.2%) |  |
| **Prior Systemic Therapy Regimens for Distant Metastases** |  |  | 0.68 |
| 0-1 | 61 (83.0%) | 63 (82.9%) |  |
| ≥2 | 15 (19.7%) | 13 (17.1%) |  |
| **Year Treated** |  |  | 0.62 |
| 2014-2016 | 45 (59.2%) | 48 (63.2%) |  |
| 2017-2018 | 31 (40.8%) | 28 (36.8%) |  |
| ≥3 | 16 (8.2%) | 7 (4.4%) |  |

Supplemental table 2. Patient characteristics within the matched cohort for dose intensity.

|  | **Number (%)** | **P** | **Median Survival (months)** | **12 month survival** |
| --- | --- | --- | --- | --- |
| **Number of Isocenters Treated** |  | 0.01 |  |  |
| 1 | 248 (69.9%) |  | 8.4 | 38.0% |
| 2 | 84 (23.7%) |  | 14.5 | 55.1% |
| 3 or more | 23 (6.5%) |  | 9.5 | 35.1% |
| **Primary Tumor ± Regional Nodes Treated** |  | 0.567 |  |  |
| Yes | 128 (36.1%) |  | 9.4 | 40.9% |
| No | 227 (63.9%) |  | 8.8 | 42.5% |
| **Bone Metastases Treated** |  | 0.26 |  |  |
| Yes | 147 (41.4%) |  | 10.4 | 46.4% |
| No | 208 (58.6%) |  | 8.4 | 38.8% |
| **Brain Metastases Treated** |  | 0.02 |  |  |
| Yes | 91 (25.6%) |  | 5.5 | 32.4% |
| No | 264 (74.4%) |  | 10.2 | 45.2% |
| **Lung Metastases Treated** |  | 0.05 |  |  |
| Yes | 26 (7.3%) |  | 18.6 | 68.7% |
| No | 329 (92.7%) |  | 8.8 | 39.7% |
| **Distant Lymph Nodes Treated** |  | 0.09 |  |  |
| Yes | 25 (7.0%) |  | 10.1 | 42.0% |
| No | 330 (93.0%) |  | 9.3 | 41.9% |
| **Other Distant Sites Treated*** |  | 0.69 |  |  |
| Yes | 25 (7.0%) |  | 8.7 | 33.4% |
| No | 330 (93.0%) |  | 9.5 | 42.5% |

* abdominal wall (6), liver (5), paraspinal mass (5), adrenal (2), leptomeningeal (2), hard palate (1), muscle (1), paranephric nodule (1), parotid (1), prophylactic cranial irradiation (1), skin (1)

Supplemental Table 3. Median and 12-month Survival Stratified by Treatment Characteristics

| **Variable** | **EQD2 ≥40 Gy** | **EQD2 <40 Gy** | **p** |
| --- | --- | --- | --- |
| **Age** |  |  | 0.62 |
| <60 | 42 (21.5%) | 38 (23.8%) |  |
| ≥60 | 153 (78.5%) | 122 (76.3%) |  |
| **Gender** |  |  | 0.89 |
| Male | 99 (50.8%) | 80 (50.0%) |  |
| Female | 96 (49.2%) | 80 (50.0%) |  |
| **ECOG performance status** |  |  | <0.001 |
| 0-1 | 124 (63.6%) | 54 (33.8%) |  |
| 2 | 50 (25.6%) | 58 (36.3%) |  |
| 3-4 | 21 (10.8%) | 48 (30.0%) |  |
| **Albumin** |  |  | <0.001 |
| ≥3.4 | 98 (50.3%) | 55 (34.4%) |  |
| 2.4 to 3.3 | 53 (27.2%) | 75 (46.9%) |  |
| <2.4 | 9 (4.6%) | 15 (9.4%) |  |
| Unknown | 35 (18.0%) | 15 (9.4%) |  |
| **Neutrophil to Lymphocyte Ratio** |  |  | 0.19 |
| ≤4.0 | 75 (38.5%) | 56 (35.0%) |  |
| >4.0 | 88 (45.1%) | 89 (55.6%) |  |
| Unknown | 32 (16.4%) | 15 (9.4%) |  |
| **Primary tumor site** |  |  |  |
| Lung | 83 (42.6%) | 69 (43.1%) |  |
| Prostate | 19 (9.7%) | 20 (12.5%) |  |
| Breast | 15 (7.7%) | 23 (14.4%) |  |
| Colorectal | 10 (5.1%) | 9 (5.6%) |  |
| Uterus | 11 (5.6%) | 6 (3.8%) |  |
| Esophagus/Gastric | 6 (3.1%) | 8 (5.0%) |  |
| Unknown Primary | 8 (4.1%) | 5 (3.1%) |  |
| Melanoma | 9 (4.6%) | 3 (1.9%) |  |
| Kidney | 8 (4.1%) | 0 (0.0%) |  |
| Pancreatic/Hepatobiliary | 3 (1.5%) | 5 (3.1%) |  |
| Other (Ovary, Cervix, Sarcoma, Skin, Bladder, Pleura, Head and Neck, Vulva) | 23 (11.8%) | 12 (7.5%) |  |
| **Favorable Primary Site** |  |  | 0.24 |
| Breast, Prostate or Kidney | 42 (21.5%) | 43 (26.9%) |  |
| Others | 153 (78.5%) | 117 (73.1%) |  |
| **Number of Active Tumors** |  |  | <0.001 |
| 1 to 5 | 92 (47.2%) | 14 (8.8%) |  |
| ≥6 | 103 (52.8%) | 146 (91.3%) |  |
| **Liver Metastases** |  |  | <0.001 |
| Yes | 165 (84.6%) | 110 (68.8%) |  |
| No | 30 (15.4%) | 50 (31.3%) |  |
| **Bone Only Metastases** |  |  | 0.99 |
| Yes | 44 (22.6%) | 36 (22.5%) |  |
| No | 151 (77.4%) | 124 (77.5%) |  |
| **Hospitalization within the Prior 3 Months** |  |  | 0.001 |
| Yes | 97 (49.7%) | 107 (66.9%) |  |
| No | 98 (50.3%) | 53 (33.1%) |  |
| **Prior Systemic Therapy Regimens for Distant Metastases** |  |  | 0.74 |
| 0-1 | 162 (83.1%) | 135 (84.4%) |  |
| ≥2 | 33 (16.9%) | 25 (15.6%) |  |
| **Number of Radiation Isocenters** |  |  | 0.05 |
| 1 | 126 (64.6%) | 122 (76.3%) |  |
| 2 | 53 (27.2%) | 31 (19.4%) |  |
| ≥3 | 16 (8.2%) | 7 (4.4%) |  |
| **Primary Tumor ± Regional Nodes Treated** |  |  | <0.001 |
| Yes | 88 (45.1%) | 40 (25.0%) |  |
| No | 107 (54.9%) | 120 (75.0%) |  |
| **Bone Metastases Treated** |  |  | <0.001 |
| Yes | 60 (30.8%) | 87 (54.4%) |  |
| No | 135 (69.2%) | 73 (45.6%) |  |
| **Brain Metastases Treated** |  |  | 0.46 |
| Yes | 53 (27.2%) | 38 (23.8%) |  |
| No | 142 (72.8%) | 122 (76.3%) |  |
| **Lung Metastases Treated** |  |  | <0.001 |
| Yes | 23 (11.8%) | 3 (1.9%) |  |
| No | 172 (88.2%) | 157 (98.1%) |  |
| **Distant Nodes Treated** |  |  | 0.08 |
| Yes | 18 (9.2%) | 7 (4.4%) |  |
| No | 177 (90.8%) | 153 (95.6%) |  |
| **Other Distant Sites Treated*** |  |  | 0.03 |
| Yes | 19 (9.7%) | 6 (3.8%) |  |
| No | 176 (90.3%) | 154 (96.3%) |  |

* abdominal wall (6), liver (5), paraspinal mass (5), adrenal (2), leptomeningeal (2), hard palate (1), muscle (1), paranephric nodule (1), parotid (1), prophylactic cranial irradiation (1), skin (1)

Supplemental Table 4. Patient and Treatment Characteristics Stratified by Radiation Dose Intensity

| **Variable** | **Hazard Ratio** | **95% Confidence Interval** | **P value** |
| --- | --- | --- | --- |
| ECOG Performance Status (0-1 vs. 2 vs. 3-4) | 1.67 | 1.38 to 2.03 | <0.001 |
| Radiation Dose Intensity (High vs. Low) | 1.97 | 1.43 to 2.71 | <0.001 |
| Serum Albumin (≥3.4 vs. 2.4 to 3.3 vs. <2.4) | 1.52 | 1.19 to 1.94 | 0.001 |
| Tumor Site (Breast, Kidney or Prostate vs. Other) | 1.69 | 1.16 to 2.47 | 0.006 |
| Liver Metastases (No vs. Yes) | 1.53 | 1.09 to 2.13 | 0.013 |
| Number of Active Tumors (1 to 5 vs. ≥6) | 1.51 | 1.05 to 2.17 | 0.026 |
| Bone Only Metastases (Yes vs. No) | 1.33 | 0.87 to 2.05 | 0.192 |
| Hospitalized within Prior 3 Months (No vs. Yes) | 1.19 | 0.86 to 1.65 | 0.295 |
| Brain Metastases Treated (No vs. Yes) | 1.18 | 0.85 to 1.64 | 0.327 |
| Number of Isocenters (>1 vs. 1) | 1.14 | 0.84 to 1.56 | 0.401 |
| Neutrophil to Lymphocyte Ratio (≤4.0 vs. >4) | 1.14 | 0.85 to 1.52 | 0.385 |
| Lung Metastases Treated (Yes vs. No) | 1.03 | 0.60 to 1.77 | 0.911 |

Supplemental Table 5. **Expanded Cox Multivariable Analysis Using Additional Treatment Variables**
